# IL-12 from endogenous cDC1, and not vaccine DC, is required for Th1 induction

**DOI:** 10.1172/jci.insight.135143

**Published:** 2020-05-21

**Authors:** DiyaaElDin Ashour, Panagiota Arampatzi, Vladimir Pavlovic, Konrad U. Förstner, Tsuneyasu Kaisho, Andreas Beilhack, Florian Erhard, Manfred B. Lutz

**Affiliations:** 1 Institute for Virology and Immunobiology, and; 2 Core Unit Systems Medicine, University of Würzburg, Würzburg, Germany.; 3 ZB MED, Information Centre for Life Sciences, Cologne, Germany.; 4 TH Köln, University of Applied Sciences, Institute of Information Science, Cologne, Germany.; 5 Department of Immunology, Institute of Advanced Medicine, Wakayama Medical University, Wakayama, Wakayama, Japan.; 6 Department of Internal Medicine II, University Hospital Würzburg, Würzburg, Germany.

**Keywords:** Immunology, Vaccines, Cytokines, Dendritic cells, Th1 response

## Abstract

Success of DC vaccines relies on the quality of antigen presentation, costimulation, lymph node migration, and the release of IL-12, in case of Th1 priming. Here, we provide evidence for interaction between the injected vaccine DCs with endogenous lymph node–resident DCs for Th1 induction. While migration of the injected DCs was essential for antigen delivery to the lymph node, the injected DCs contributed only partially to Th0 priming and were unable to instruct Th1 generation. Instead, we provide evidence that the lymph node–resident XCR1^+^ DCs are activated by the injected DCs to present the cognate antigen and release IL-12 for Th1 polarization. The timing of interactions in the draining lymph nodes appeared step-wise as (a) injected DCs with cognate T cells, (b) injected DCs with bystander DCs, and (c) bystander DCs with T cells. The transcriptome of the bystander DCs showed a downregulation of Treg- and Th2/Th9-inducing genes and self-antigen presentation, as well as upregulation of MHC class II and genes required for Th1 instruction. Together, these data show that injected mature lymph node migratory DCs direct T cell priming and bystander DC activation, but not Th1 polarization, which is mediated by endogenous IL-12p70^+^XCR1^+^ resident bystander DCs. Our results are of importance for clinical DC-based vaccinations against tumors where endogenous DCs may be functionally impaired by chemotherapy.

## Abstract 2

Successful Th1 priming by DC vaccines in mice depends on IL-12 from endogenous and XCR1^+^ cDC1 population.

## Graphical abstract

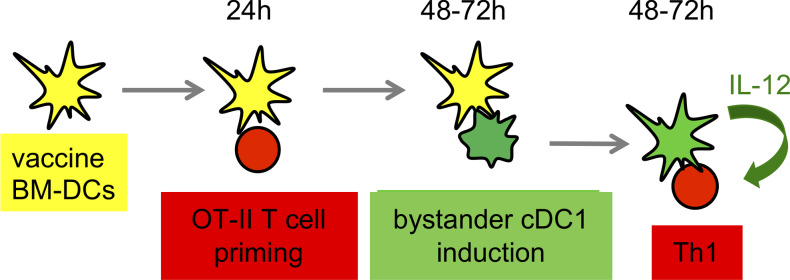

## Introduction

DCs play a central role in priming and polarization of naive CD4^+^ T cells into subsequent Th subsets (1). DCs are composed of defined subsets with different ontogenies, surface markers, functions, and spatial organization within lymphoid organs (2–4). Plasmacytoid DCs (pDCs) and 2 subsets of conventional DCs (cDCs) can act tolerogenic (5–8) but also as sentinels for pathogen recognition for subsequent CD4^+^ T cell priming and polarization toward Th1, Th2, or Th17 helper responses, depending on the subset and the pathogen-directed signals (9, 10).

pDCs are a major source of IFN-α for antiviral responses (11), while cDCs are classified into XCR1^+^ cDC1 and CD11b^+^ cDC2 (2, 3). cDC2s have been further subdivided into ESAM^lo^CD11b^+^ cDC2 that are dependent on Klf4 transcription and functionally induce Th2 responses (12), while ESAM^hi^CD11b^+^ cDC2 are dependent on the Notch2 transcription factor and preferentially induce Th17 responses (13). The CD8^+^ or CD103^+^ XCR1^+^ cDC1 subset is specialized in MHC class I–dependent (MHC-I–dependent) cross-presentation for CD8^+^ T cell responses (14), as well as in CD4^+^ Th1 polarization against intracellular pathogens and tumors (10).

Under inflammatory conditions, another subset of DCs of monocytic origin (MoDC) is generated that are characterized by expression of macrophage-related markers CD64^+^CD11b^+^MAR1^+^ (15). MoDCs represent a reservoir for rapid recruitment of DCs during inflammation (16). In vitro GM-CSF generated BM-derived DCs (BM-DCs) resemble MoDCs (17) and such BM-DCs can be instructed by pathogens or inflammatory signals to induce Th1, Th2, and Th17 responses, depending on the quality and magnitude of the stimulation (18, 19).

The induction of Th1 responses by DCs relies on 3 distinct stimuli from DCs. MHC peptide complexes ligating the TCR (signal 1) and CD80/CD86 costimulation (signal 2) lead to T cell activation and proliferation, reaching a stage termed Th0, where IL-2 production by the T cells can be measured, but without secretion of Th1 cytokines such as IFN-γ (20). Signal 3 has been proposed for the induction of polarized CD4^+^ Th responses (21), and the heterodimeric IL-12p70 cytokine turned out to be critical for the induction of Th1 cells (22). IL-12 production by DCs can be induced by different pathogen signals but not proinflammatory cytokines (23).

Human MoDCs can be derived from CD14^+^ peripheral blood monocytes (24) and are the prime candidates for adoptive DC vaccine trials in tumor patients (25). Still, the most common DC vaccination approach is based on MoDCs (26, 27), and they are typically matured by a cytokine cocktail consisting of IL-1β/TNF-α/IL-6/PGE_2_ (28). Although tumor therapy with cocktail-matured MoDCs has proven to be successful in melanoma patients, it is still unclear how such cytokine cocktail–matured MoDCs that are unable to produce IL-12 (29) are readily able to induce Th1 responses in these patients (27, 29, 30). Also, the use of BM-DC from IL-12–deficient mice for vaccination against *Leishmania* infection indicated that the development of Th1 responses relied on an undetermined source of IL-12 production by the recipient mice, not the injected DCs (31). These findings force researchers to question the common belief that injected vaccine MoDCs do provide signals 1, 2, and 3 for Th1 priming. In fact, it has been proposed that CCR7-dependent MoDC migration and production of IL-12 are mutually exclusive functions (32). We have shown before that injected BM-DCs reaching the draining lymph node lack IL-12 production, and they rather induce cytokine production by host endogenous DCs (33), suggesting transfer of Th1-instructing information to endogenous DCs. Indeed, cooperation between pDCs and cDC subsets can improve antiviral CD8^+^ T cell immune responses, although IL-12 production was not investigated (34). Although there is ample evidence for endogenous bystander IL-12 production, the endogenous IL-12 source for Th1 induction has not been identified.

In this study, we generated a chimeric situation by injection of different gene-modified BM-DCs into different strains of gene-modified recipient mice. This allowed us to identify the separate functional contributions of injected versus endogenous DCs for Th1 polarization. We identified the cellular source of IL-12p70 production after s.c. BM-DC vaccination as endogenous resident XCR1^+^ bystander DCs in the skin draining lymph nodes. DC-DC and DC–T cell interaction studies revealed a time course of Th0 priming by injected BM-DCs, followed by antigen transfer and bystander activation of the IL-12^+^XCR1^+^ bystander DCs by BM-DCs, and finally IL-12^+^XCR1^+^ bystander DC interactions for Th1 induction. Transcriptional profiling of the bystander DCs underscores their Th1 polarization potential. This study shows that DC vaccination requires the bystander activation of endogenous DCs for Th1 priming.

## Results

### IL-12p70 production by injected OVA-loaded BM-DCs is not required for Th1 polarization.

**Figure 1:**
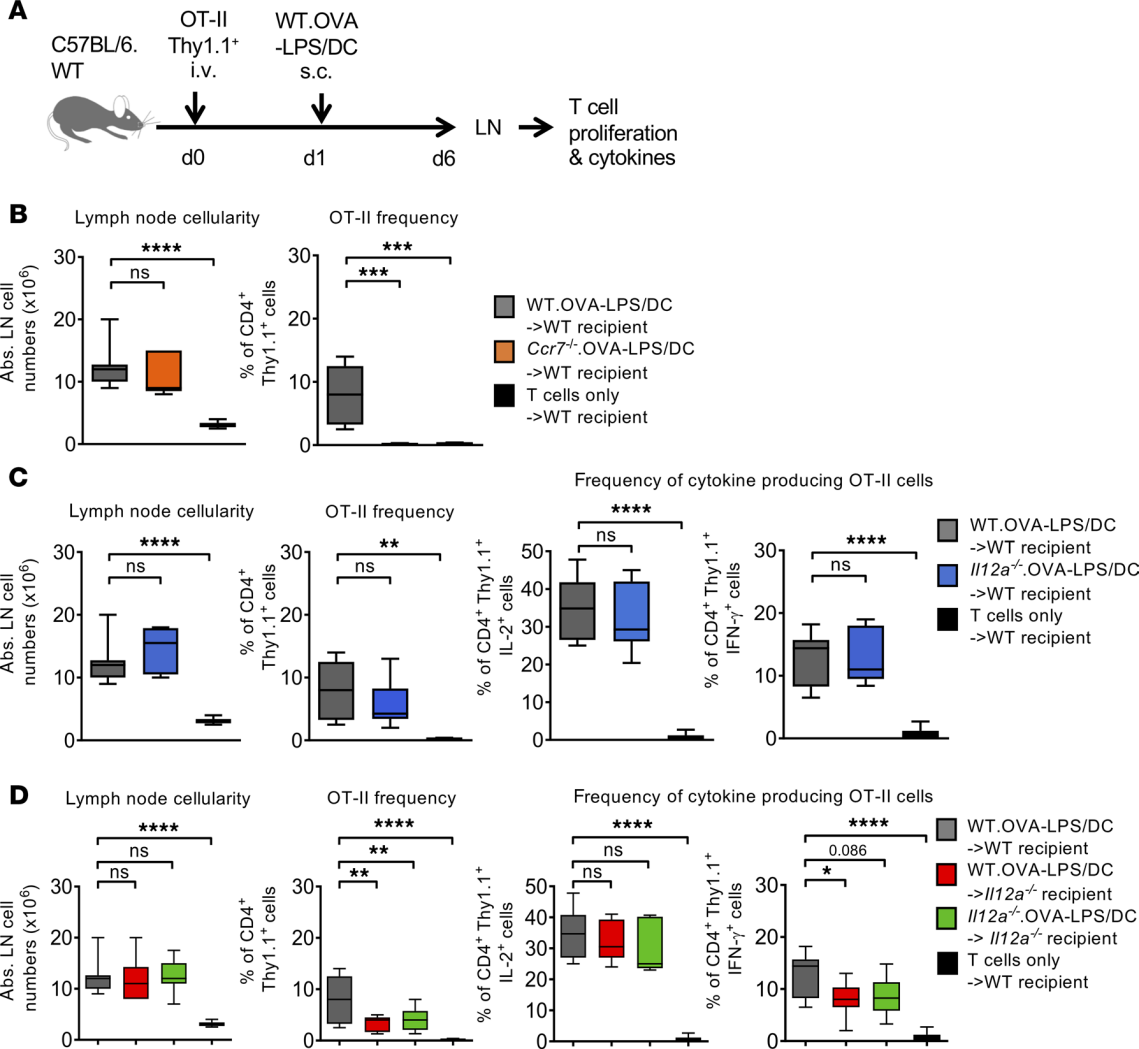
Efficient DC vaccination IL-12p70 production by endogenous DCs rather than injected BM-DCs. (**A**) OT-II^+^Thy1.1^+^ T cell priming analyzed after 6 days of OVA-loaded (10 μM), LPS-matured (0.5 μg/mL) BM-DC (WT.OVA-LPS/DC) s.c. footpad injection. (**B**) Graphs comparing lymph node cell counts and frequency of injected OT-II^+^Thy1.1^+^CD4^+^ T cells after s.c. injection of WT.OVA-LPS/DC (gray bars) or *Ccr7*^–/–^ OVA-LPS/DC (orange bars) compared with T cell injection alone (black bars). (**C**) Graphs comparing lymph node cell counts, frequency of injected OT-II^+^Thy1.1^+^CD4^+^ T cells, and percentage of the cytokine-producing cells after s.c. injection of WT.OVA-LPS/DC (gray bars) or *Il12a^–/–^* OVA-LPS/DC (blue bars) both into C57BL/6 WT recipient mice compared with T cell injection alone (black bars). (**D**) Graphs comparing lymph node cell counts, frequency of injected OT-II^+^Thy1.1^+^CD4^+^ T cells, and percentage of the cytokine=producing cells after s.c. injection of WT.OVA-LPS/DC into WT recipient mice (gray bar), WT.OVA-LPS/DC into *Il12a*^–/–^ recipient mice (red bars), and *Il12a*^–/–^ OVA-LPS/DC into *Il12a*^–/–^ recipient mice (green bars), compared with T cell injection alone (black bars). Data are representative of 3 independent experiments analyzing at least 5 mice per group. One-way ANOVA with multiple comparisons and Tukey’s post hoc test; *P < 0.05, **P < 0.01, ***P < 0.005, ****P < 0.001.

To address whether the injected DCs are capable of providing all 3 signals for the priming, proliferation, and polarization of antigen-specific CD4^+^ T cells toward a Th1 response, we used BM-DCs as a source of MoDCs (17). Following the i.v. injection of CellTrace Violet–labeled (CTV-labeled) OT-II^+^Thy1.1^+^ cells in mice with a Thy1.2 background, OVA-loaded BM-DCs that were matured with LPS (OVA-LPS/DC) were injected into footpads to induce a Th1 response in the popliteal skin draining lymph node. BM-DCs were detected by their fluorescence label between 24 and 72 hours after injection but disappeared after 6 days (Supplemental Figure 1; supplemental material available online with this article; https://doi.org/10.1172/jci.insight.135143DS1). T cell proliferation and cytokine production were analyzed at day 6 (d6) (Figure 1A). We tested whether migration of the injected BM-DCs to the popliteal lymph nodes is required for antigen presentation. OVA-LPS/DCs were generated from migration-deficient *Ccr7*^–/–^ mice, as shown before (35). The results indicate that the lymph nodes were enlarged by the *Ccr7*^–/–^ BM-DC injection, similar to *Ccr7*^+/+^ BM-DC injections, clearly indicating inflammation in the lymph node. However, no T cell expansion was observed in *Ccr7*^–/–^ BM-DC injected animals (Figure 1B). As a control, OT-II^+^Thy1.1^+^CD4^+^ T cells did not expand without BM-DC injection, and lymph node size did not increase (Figure 1B). This indicated that the migration of injected BM-DCs to the lymph node is required for T cell expansion. It also excludes that nonmigratory BM-DCs hand over antigen at the injection site to endogenous DCs that take over or add to the T cell priming.

To test whether the injected BM-DCs also directly provide the Th1 polarizing IL-12 signal, LPS-matured OVA-loaded BM-DCs were injected and T cell cytokines were measured by intracellular FACS analysis. OT-II^+^Thy1.1^+^CD4^+^ T cells induced IFN-γ and IL-2 production, indicative of a Th1 polarization (Figure 1C). Surprisingly, a similar cytokine response was observed when the injected OVA-LPS/DCs were generated from mice lacking the p35 subunit (*Il12a*) of the IL-12p70 heterodimer. No significant reduction in lymph node cellularity and in OT-II^+^Thy1.1^+^CD4^+^ T cell expansion (Figure 1C) or in IFN-γ and IL-2 production (Figure 1C) was observed following *Il12a*^–/–^ BM-DC injection. Together, our data are in agreement with previous reports (27, 31) indicating that IL-12p70 production by injected BM-DCs is not required for CD4^+^ T cell priming and Th1 polarization. It remains to be determined whether another cellular source can provide IL-12 to direct Th1 polarization.

### IL-12p70 production by endogenous cells is required for Th1 polarization.

Since IL-12p70 production by the injected BM-DCs was not needed to generate a Th1 response, we tested whether an endogenous cellular source can provide IL-12p70. To test this, we injected either WT or *Il12a*^–/–^ BM-DCs into *Il12a*^–/–^ recipient mice after OT-II^+^Thy1.1^+^ injection, as described before. On d6, lymph node cellularity increased in all groups, similar to when using WT recipients. However, the percentage OT-II^+^Thy1.1^+^CD4^+^ T cells was significantly reduced in *Il12a*^–/–^ recipient mice (Figure 1D). This may indicate that BM-DC–derived p35 could contribute directly or indirectly to the general expansion of antigen-specific T cells in the draining lymph node.

Analyzing the cytokine production of the OT-II cells in the same experimental settings, the frequency of IL-2–producing T cells remained unchanged, while the percentage of IFN-γ producers significantly declined (Figure 1D). These data indicate that, in the absence of endogenous IL-12p35, the priming of T cells by injected BM-DCs remains intact and the antigen-specific CD4^+^ T cells develop into an intermediate IL-2^+^IFN-γ^–^ Th0 phenotype. The reduced frequency of OT-II T cells in IL-12p35–deficient mice also indicates that the function of IL-12 goes beyond Th1 instruction. Polarization into Th1 cells requires an endogenous cellular source for IL-12p70. While BM-DC–derived IL-12p35 does not play a role for Th1 polarization, it may contribute by indirect effects on inflammation and T cell expansion.

### MHC-II^hi^CD103^+^ cDC1s are the major source of IL-12p40 in the draining lymph node.

**Figure 2:**
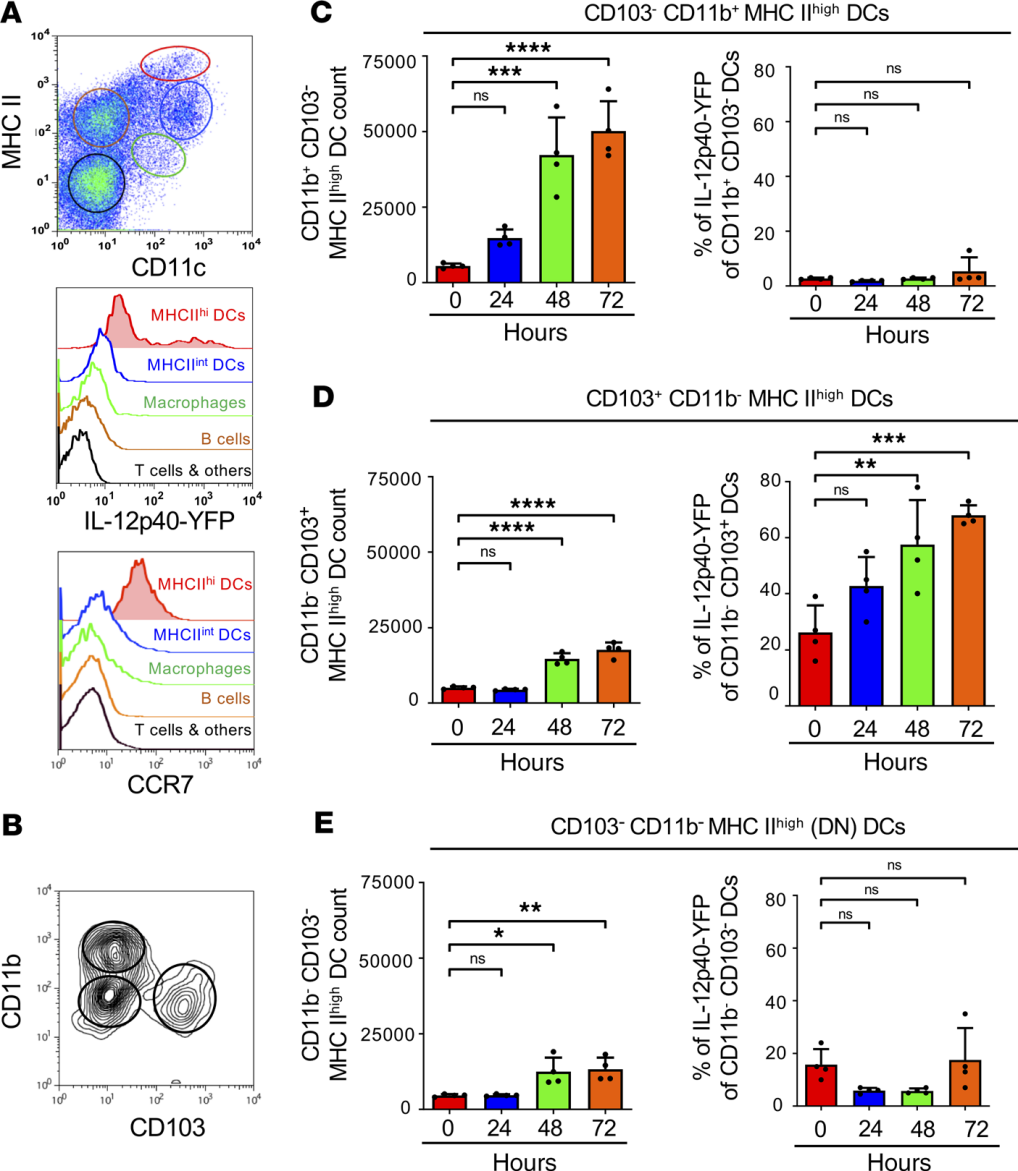
Endogenous MHC-II^hi^CD103^+^XCR1^+^Langerin^+^CD11b^lo^ DCs are the main producers of IL-12p40 after DC vaccination. (**A**) Representative flow cytometry plot of popliteal lymph nodes subpopulations gated based on their expression of CD11c and MHC-II in a *Yet40* reporter mouse (upper panel) and histogram plots of IL-12p40–YFP production and CCR7 expression of each subpopulation (lower panels). (**B**) Gating strategy to identify CD11c^+^MHC-II^hi^ DC subsets. (**C–E**) Graphs showing absolute counts of CD11c^+^MHC-II^hi^ DCs (left panels) and percentage of IL-12p40–YFP producing cells from CD11b^+^ dermal DCs (**C**) CD103^+^ dermal DCs (**D**) and DN DCs (**E**) (right panels) after s.c. injection of WT.LPS/DC into *Yet40* recipient mice (24-, 48-, 72-hour time points). Data are representative of 3 independent experiments analyzing at least 5 mice per group. One-way ANOVA with multiple comparisons and Tukey’s post hoc test; *P < 0.05, **P < 0.01, ***P < 0.005, ****P < 0.001.

To identify the endogenous cell subset responsible for the production of the third signal for Th1 response, we made use of Yet40 mice that express YFP under control of the promoter of the p40 subunit of IL-12 (36). In the skin draining lymph nodes of untreated healthy mice, we noticed that the only IL-12p40–YFP–producing cells were CD11c^int^MHC-II^hi^ cells that also expressed CCR7 (Figure 2A). The IL-12p40^+^ population corresponds to the CCR7^+^ tolerogenic steady state migratory DCs (5, 37). The constitutive expression of IL-12p40 at the steady state was observed previously, and it was suggested that the formation of p40 homodimers has an antagonistic effect on IL-12p70 (38). A significant increase in IL-12p40–YFP production above this basal level was considered as proinflammatory IL-12p40, required for the production of Th1-inducing IL-12p70. We then further analyzed the subsets gated for CD103^+^CD11b^–^ cDC1, CD103^–^CD11b^+^ cDC2, and the double negative (DN) DCs for both markers within the CD11c^int^MHC-II^hi^ population (Figure 2B). Within the CD11c^int^MHC-II^hi^ DC gate, the CD103^+^ CD11b^–^ cDC1 subset was the main source for steady state IL12-p40–YFP production (Figure 2D, right panel).

To test for bystander production of IL-12 after DC vaccination, eFluor 670–labeled/LPS-matured BM-DCs were injected s.c. into the flank or footpad of Yet40 mice, and the YFP production in the inguinal or popliteal lymph nodes, respectively, was analyzed after 24, 48, and 72 hours. eFluor 670 labeling of injected BM-DCs was used to exclude them from the analyses of the endogenous DC subsets. All 3 DC subsets increased their frequency in the draining lymph nodes in response to the injection-induced inflammation (Figure 2, C–E, left panels). Only the CD103^+^XCR1^+^CD11b^–^ cDC1 subset showed a significant increase in IL-12p40–YFP production in a time-dependent fashion (Figure 2D, right panel). No YFP production was observed by CD11b^+^CD103^–^ cDC2s (Figure 2C, right panel). CD11b^–^CD103^–^ (DN) DCs showed substantial YFP production under steady-state conditions, which significantly dropped 24 and 48 hours after BM-DC injection (Figure 2E, right panel). Although only IL-12p40 was analyzed here, these data suggest that CD103^+^ cDC1s were the major candidate for bystander IL-12p70 production for Th1 polarization after BM-DC injection.

### CCR7-independent resident DCs provide the third signal for Th1 polarization and contribute to antigen presentation.

**Figure 3:**
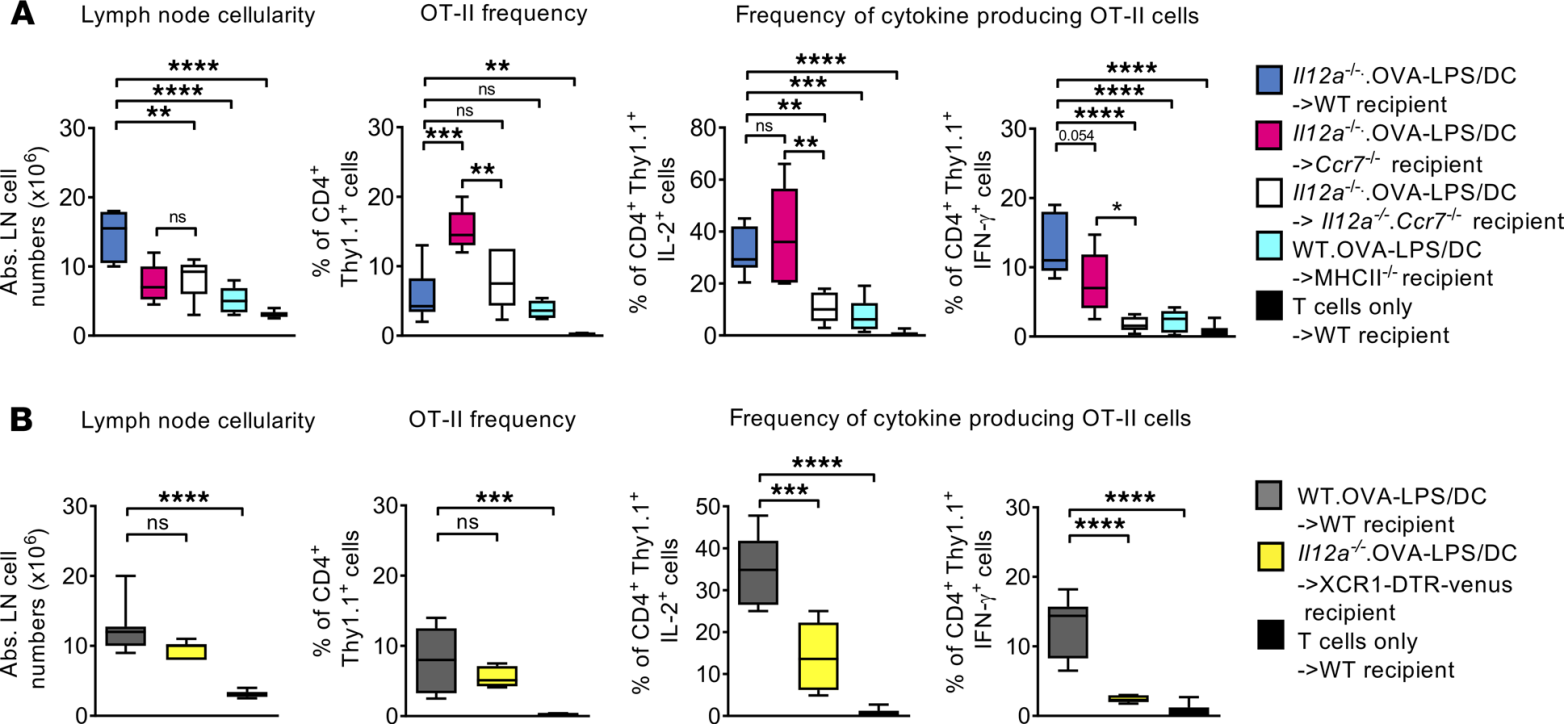
Endogenous CD103^+^XCR1^+^ resident DCs are required for antigen presentation and Th1 polarization. (**A**) Graphs comparing lymph node cell counts, frequency of injected OT-II^+^Thy1.1^+^CD4^+^ T cells, and percentage of the cytokine-producing cells after s.c. injection of *Il12a*^–/–^ OVA-LPS/DC into WT mice (blue bars), *Il12a*^–/–^ OVA-LPS/DC into *Ccr7*^–/–^ recipient mice (red bars), *Il12a*^–/–^ OVA-LPS/DC into *Il12a^–/–^*Ccr7^–/–^ mice (white bars), or WT.OVA-LPS/DC into *MHC-II^–/–^* (turquoise bars) recipient mice compared with T cell injection alone (black bars). (**B**) Graphs comparing lymph node cell counts, frequency of injected OT-II^+^Thy1.1^+^CD4^+^ T cells, and percentage of the cytokine-producing cells after s.c. injection of WT.OVA-LPS/DC into WT recipient mice (gray bar) or *Il12a^–/–^* OVA-LPS/DC into XCR1-DTR-Venus recipient mice (yellow bars) compared with T cell injection alone (black bars). Data are representative of 2 independent experiments analyzing at least 5 mice per group. One-way ANOVA with multiple comparisons and Tukey’s post hoc test; *P < 0.05, **P < 0.01, ***P < 0.005, ****P < 0.001.

The CD11c^int^MHC-II^hi^ population is composed mainly of migratory DCs, as described before (5). However, resident CD11c^hi^MHC-II^int^ DCs increase their MHC-II expression when they mature and, thus, enter this gate. To identify whether the CD11c^int^MHC-II^hi^ endogenous DC subset required for Th1 polarization after BM-DC injection is a migratory or a resident one, we used *Ccr7*^–/–^ recipient mice that lack endogenous migratory DCs. These mice were injected with BM-DCs generated from *Il12a*^–/–^ animals. While the lymph node cellularity decreased, the frequency of OT-II cells was increased (Figure 3A), but no significant reduction in the frequency of IL-2 and only a trend for IFN-γ–producing T cells was observed when compared with the WT recipients injected with *Il12a^–/–^* DCs (Figure 3A). This indicates that endogenous CCR7^+^ migratory DCs of the recipient mouse do not significantly contribute to Th1 polarization after BM-DC injection. On the other hand, injecting *Il12a*^–/–^ BM-DCs into *Il12a*^–/–^*Ccr7*^–/–^ recipient mice showed comparable values for lymph node cellularity and OT-II frequency compared with injection of *Il12a*^–/–^ BM-DCs into WT mice, but there was a strong drop in IL-2–producing T cells and basically a complete loss of IFN-γ producers, indicating a residual Th0 response (Figure 3A). These findings point to a resident CCR7^–^ DC subset as the main producers of IL-12 required for Th1 polarization.

The question remained whether the endogenous DCs only deliver IL-12 or also contribute to antigen presentation. To test this, we used MHC-II–deficient mice as recipients and injected WT BM-DCs. Due to a general lack of CD4^+^ T cells in these mice, the lymph node cellularity was significantly reduced compared with WT recipient mice (Figure 3A). Similar to the IL-12 deficiency of recipient mice, the lack of MHC-II antigen presentation also dramatically affected OT-II frequencies and reduced the frequencies of IL-2^+^ and IFN-γ^+^ T cells (Figure 3A). These results suggest that endogenous resident DCs substantially contribute to antigen presentation.

To this point, the results indicate that endogenous lymph node–resident CCR7^–^CD103^+^ cDC1s represent the bystander DC population that is producing IL-12 for the Th1 polarization of OT-II cells. The bystander cDC1 also substantially contributes to antigen presentation, suggesting an OVA antigen transfer from the injected BM-DCs to the bystander cDC1s.

### XCR1^+^CD103^+^ cDC1s provide the third signal for Th1 induction after BMDC injection.

So far, all evidence points to CCR7^–^CD103^+^ cDC1s as endogenous IL-12 producers for Th1 polarization in the BM-DC injection setup. To conclusively show the critical role of cDC1s for Th1 polarization, we used XCR1-DTR-Venus mice, where the XCR1^+^CD103^+^ cDC1 subset can be conditionally depleted by diphtheria toxin (DTX) injection (39). The mice were injected with OT-II^+^Thy1.1^+^ cells followed by *Il12a*^–/–^ OVA-LPS/DC injection as described before; then XCR1^+^ DCs were depleted using DTX. In these mice, there was a slight reduction in lymph node cellularity at d6 after BM-DC injection, while CD4^+^OT-II^+^Thy1.1^+^ T cells expanded similar to WT mice (Figure 3B). The production of IL-2, however, was significantly reduced, indicating an impairment in T cell priming, and IFN-γ production was completely lost (Figure 3B). These results conclusively show the requirement of XCR1^+^CD103^+^ cDC1s as the critical bystander DC subset to allow Th1 polarization after BM-DC vaccination.

### YFP^+^ endogenous DCs communicate with injected BM-DCs at 48–72 hours.

The contribution of bystander DCs to antigen presentation suggests antigen transfer from injected BM-DCs that would require their interaction in the draining lymph nodes. One possibility of DC-DC interaction leading to bystander activation is that LPS bound to TLR4 on the migrated BM-DCs is presented to other DCs in the lymph node. In vitro data indicate that LPS-matured BM-DCs can activate cocultured immature BM-DCs to secrete IL-12p40, while CpG-matured BM-DCs were unable to show this effect (Supplemental Figure 2). These data suggest that LPS remains bound to surface TLR4 and can be presented to bystander DCs, while CpG seems to be efficiently internalized by DEC-205/CD205 to bind TLR9 within intracellular vesicles (40). Since DEC-205 is expressed specifically on XCR1^+^ cDC1s, as detected by the NLDC-145 antibody (41), we hypothesized that injection of CpG-matured BM-DCs should abrogate bystander Th1 polarization in vivo. However, the use of CpG-matured, OVA-loaded BM-DCs (OVA-CpG/DC) provoked a similar lymph node swelling, OT-II^+^Thy1.1^+^CD4^+^ T cell expansion and Th1 frequencies, as observed after OVA-LPS/DC injection, while the frequency of IL-2^+^ OT-II cells was slightly reduced (Supplemental Figure 3). These data indicate that injected BM-DCs possess additional mechanisms of bystander DC activation beyond the presentation of surface-bound pathogen.

**Figure 4:**
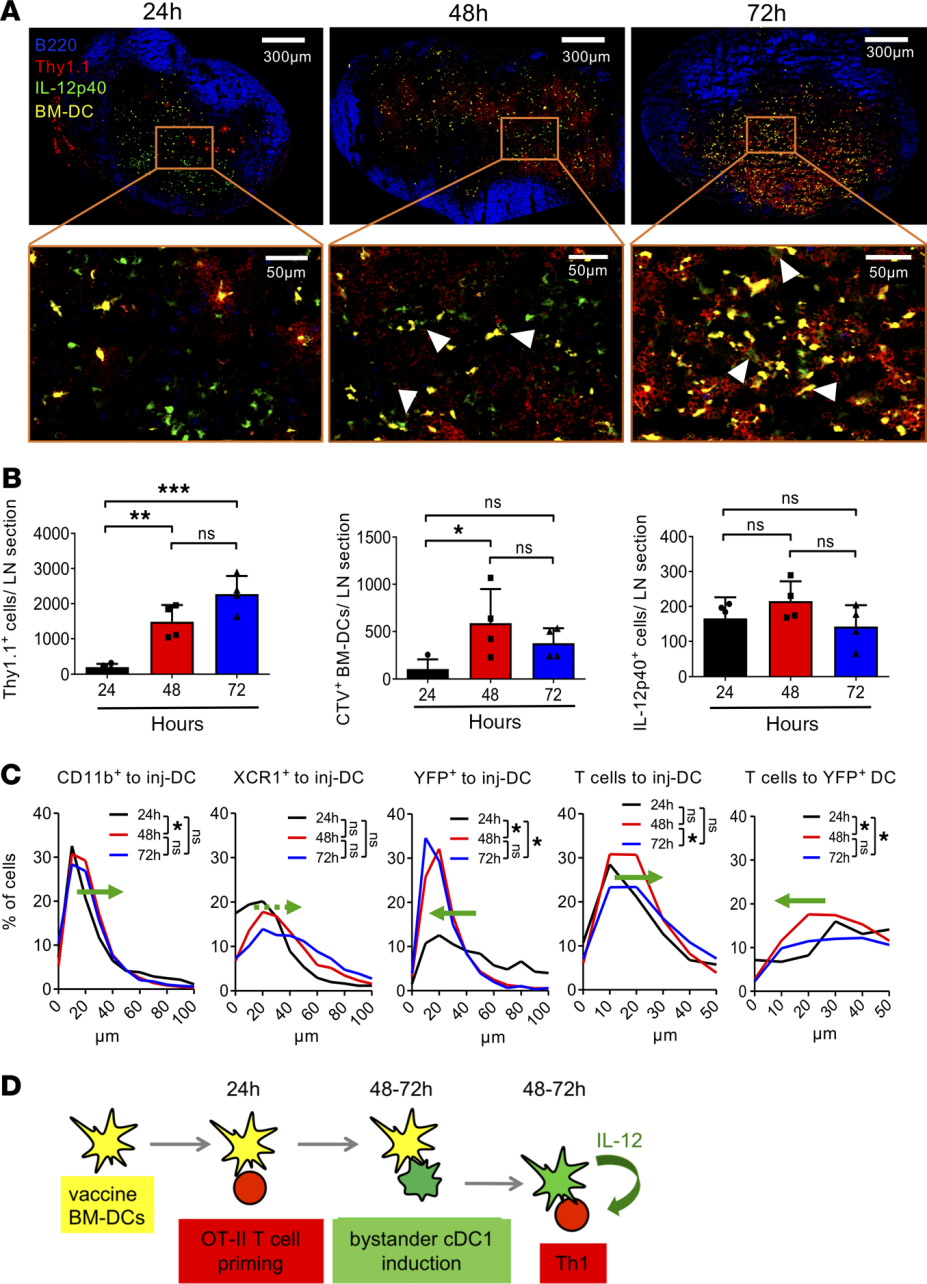
YFP^+^XCR1^+^ DCs interact with injected DCs and antigen-specific T cells at later time points. (**A**) Representative immunofluorescence microscopy images of whole popliteal lymph nodes sections (upper row) and magnification of the T cell area after of OT-II^+^Thy1.1^+^ T cell injection (red) + CTV-labeled *Il12a^–/–^* OVA-LPS/DC s.c. injection (yellow) into IL-12p40–YFP mice (green cells) (24, 48, or 72 hours after injection). White arrowheads point to the points of interaction between injected yellow DCs and YFP endogenous green DCs. (**B**) Graphs showing number of OT-II^+^Thy1.1^+^ T cells, CTV-labeled OVA-LPS/DC, and YFP^+^ endogenous DCs/popliteal lymph node cut 24, 58, 72 hours after DC injection. (**C**) Graphs showing the relative distance of CD11b^+^ cells, XCR1^+^ cells, and YFP^+^ cells to CTV-labeled OVA-LPS/DC in the peripheral lymph nodes 24, 48, or 72 hours after DC injection. Graphs showing the relative distance of OT-II^+^Thy1.1^+^ T cells to CTV-labeled OVA-LPS/DC or to YFP^+^ endogenous DCs in the peripheral lymph nodes 24, 48, or 72 hours after DC injection. Green arrow indicates the distance shift at 48 and 72 hours compared with 24 hours. Data are representative of 2 independent experiments analyzing at least 4 mice per group. One-way ANOVA with multiple comparisons and Tukey’s post hoc test; ***P < 0.0001, **P < 0.001, *P < 0.05. (**D**) Model about the time kinetics of cellular interactions suggested by the microscopic analyses.

To study potential DC-DC and DC–T cell communication patterns in the lymph node, OT-II^+^Thy1.1^+^ T cells were injected i.v. and CTV-labeled LPS–BM-DCs generated from *Il12a*^–/–^ mice were injected the next day into the footpads of the mice. The interaction between the injected T cells, BM-DCs, and YFP^+^ endogenous DCs was analyzed 24, 48, and 72 hours after their injection within the T cell area of popliteal draining lymph nodes by confocal microscopy. We observed distinct clusters of OT-II^+^Thy1.1^+^ cells with CTV-labeled BM-DCs within the T cell area at the 24-hour time point, as described before (42, 43). During this period, T cells form long-lasting stable conjugates with DCs (Figure 4A). At 48 hours, the presence of CTV-labeled BM-DCs peaked in the lymph nodes and declined thereafter (Figure 4B). OT-II^+^Thy1.1^+^ T cell expansion was observed peaking at 72 hours (Figure 4B). The number of IL-12p40^+^ cells remained constant, showing only a trend of higher frequencies at 48 hours (Figure 4B). We then analyzed the change in relative distance of YFP^+^ endogenous DCs to the injected CTV-labeled BM-DCs and compared it with the relative distance between the total XCR1^+^ endogenous cDC1s, which includes the presumed bystander-activated CD103^+^XCR1^+^ cDC1s and the injected BM-DCs; as a control, the distance between the unrelated CD11b^+^ cDC2s and the injected BM-DCs was measured. The relative distance between CD11b^+^ and total XCR1^+^ endogenous DCs to injected BM-DCs showed no signs of approximation but rather increased at the 48-hour and 72-hour time points when compared with the 24-hour time point (Figure 4C, green arrow directions). This is attributed to their random movement in the lymph node that gets enlarged at the 48-hour and 72-hour time point. When the relative distance between the specifically IL-12p40–producing YFP^+^ endogenous XCR1^+^ cDC1s and the injected BM-DCs was measured, a significant reduction of their distance at both 48-hour and 72-hour time points was observed when compared with 24 hours (Figure 4C, green arrow directions). These data indicate that injected BM-DCs communicate with endogenous XCR1^+^IL-12p40^+^ cDC1s in the lymph node paracortex areas to mediate the observed Th1 induction between 48 and 72 hours.

### Cognate T cells show early communication with injected BM-DCs and later with YFP^+^ endogenous DCs.

If the YFP^+^ endogenous DCs are indeed receiving signals from the injected BM-DCs at later time points, we expected them to provide the IL-12 signal to T cells later, during the T cell expansion phase. This was already indicated by the fact that IL-12p40–YFP peaked at 72 hours (Figure 2C), indicating its requirement later in the T cell expansion phase. To confirm that, we measured the relative distances of OT-II^+^Thy1.1^+^ T cells to either injected BM-DCs or YFP^+^ endogenous DCs. The relative distance between T cells and BM-DCs showed a tendency to increase after 48 hours and significantly at 72 hours compared with the 24-hour time point, possibly due to the increase in lymph node size or the termination of antigen presentation. On the other hand, the relative distance between T cells and YFP^+^ endogenous DCs was significantly reduced after 48 hours and 72 hours, as compared with the 24-hour time point (Figure 4C).

These findings suggest a coordinated timing of interactions between 2 DC types and the cognate T cells as sequential steps — first, between the injected BM-DCs and cognate T cells; second, the BM-DCs with YFP^+^XCR1^+^ endogenous bystander cDC1s; and third, YFP^+^XCR1^+^IL-12p40^+^ endogenous bystander cDC1s with the primed Th0 cells for further polarization into Th1 cells (Figure 4D).

### Transcriptional profiling of the endogenous XCR1^+^ bystander cDC1s.

**Figure 5:**
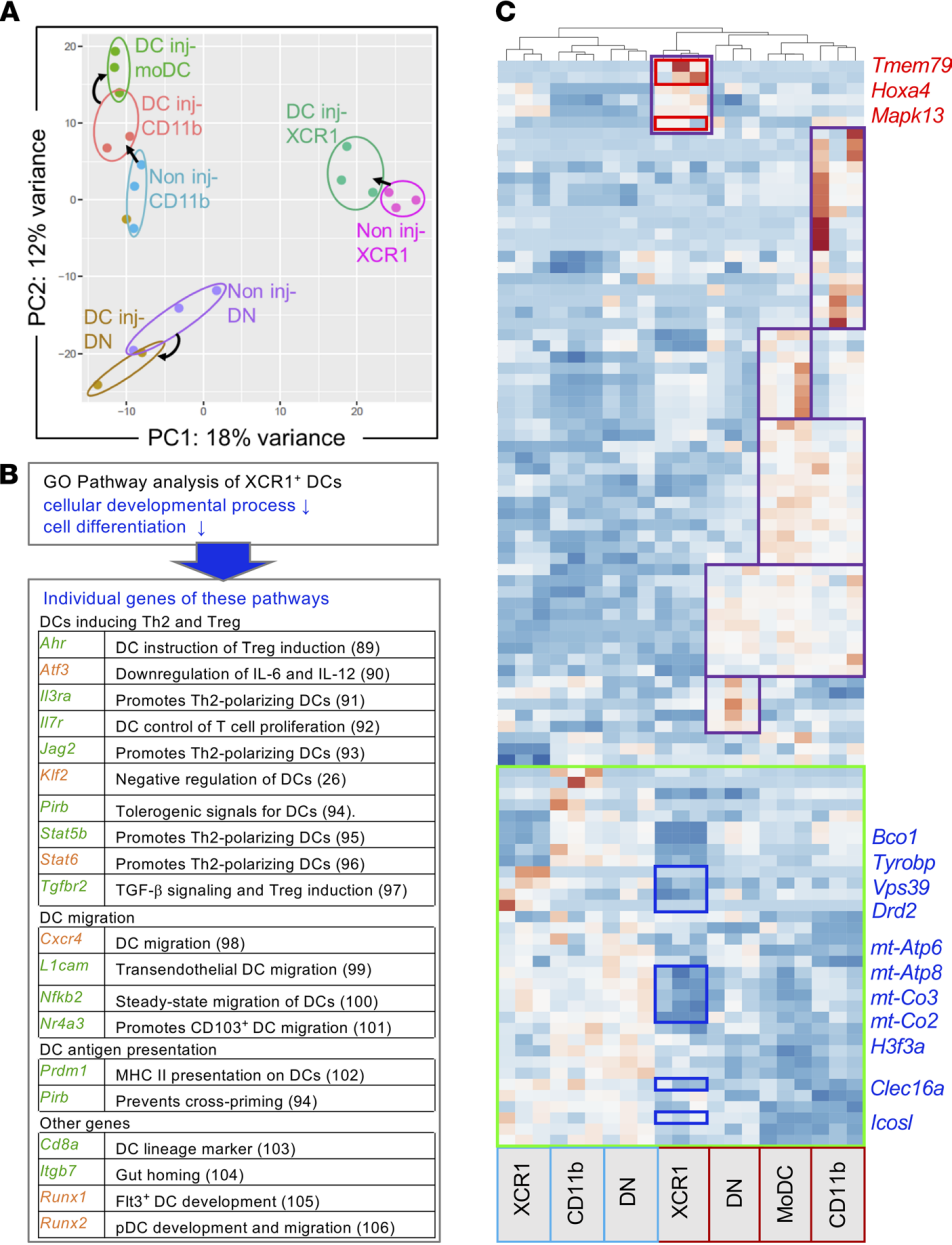
Endogenous migratory DC subsets have distinct transcriptional changes after bystander activation by BM-DC injection. (**A**) PCA for XCR1^+^, CD11b^+^, and XCR1^–^CD11b^–^ endogenous CD11c^int^MHC-II^hi^ DCs from popliteal lymph nodes before and 48 hours after immunization, and CD11b^+^CD64^+^Ly6C^–^ MoDCs compared with CD11b^+^ DCs after immunization. (**B**) Genes downregulated in XCR1^+^ DCs 48 hours (*P* < 0.05) after immunization according to the GOrilla analysis tool. Green color, down in XCR1^+^ DCs only; orange color, down in XCR1^+^ DCs and MoDCs (89–106). (**C**) Heatmap of the 112 genes that are at least 1.5-fold differentially expressed in one comparison (red, upregulated; blue, downregulated). Plotting was done using Clustvis web tool; clustering was performed using Pearson’s correlation and average linkage.

Since the IL-12^+^CD103^+^XCR1^+^ bystander cDC1 are recruited from the lymph node–resident population but IL-12p40^+^ cells are only detectable among MHC-II^hi^–expressing DCs (Figure 2C), the immature resident bystander DC population underwent maturation/activation by the DC vaccination process. To study the transcriptional changes occurring in the endogenous matured bystander cDC1, we sorted the MHC-II^hi^CD11c^+^ lymph node DC subsets before (naive mice) and 48 hours after BM-DC injection. Therefore, we could compare MHC-II^hi^XCR1^+^CD11b^–^ dermal DCs (cDC1s), MHC-II^hi^CD11b^+^XCR1^–^ dermal DCs (cDC2s), and MHC-II^hi^CD11b^–^XCR1^–^ DCs (DN) at steady-state and 48 hours after LPS–BM-DC injection. Also, the appearance of MHC-II^hi^CD11b^+^CD64^+^Ly6C^lo^ inflammation–induced MoDCs was identified, and these cells were also sorted at 48 hours. Since MoDCs could not be detected in naive mice, we were lacking a direct related control for this population. RNA sequencing (RNA-seq) was performed on 100 sorted cells from each population. Principal component analysis (PCA) segregated the samples into 3 distinct groups. Each of the 3 cDC subsets clustered differently and shifted to a different direction after their bystander activation. MoDCs clustered close to CD11b^+^ cDC2s and appeared further distant from bystander-activated CD11b^+^ cDC2s, indicating a close relation (Figure 5A). This may indicate that the general inflammatory situation in the size-expanded lymph node signals to all endogenous DC subsets.

Gene ontology (GO) and pathway enrichment analysis (http://geneontology.org/docs/go-enrichment-analysis/) for the differentially regulated genes in the XCR1^+^MHC-II^hi^ cDC1 subset indicated a downregulation of nucleosome organization, cellular development, and cellular differentiation pathways (Figure 5B). Interestingly, genes promoting TGF-β signaling and Treg induction, or Th2 induction by DCs, were included in these downregulated pathways, together with genes involved in DC migration and genes that modulate the self-antigen presentation capacity of DCs (Figure 5B).

Then, we compared the genes differentially regulated in the 4 MHC-II^hi^ DC subsets of BM-DC–injected mice with the 3 steady state MHC-II^hi^ DC subsets. A total of 112 genes were significantly up- or downregulated in at least one of the comparisons. Distinct clusters of regulated genes were observed for each DC subset (Figure 5C). Mapk13, which is involved in cytokine production; Tmem79, which is a transmembrane protein involved in the lamellar granules secretory system and skin barrier function and which might be involved in cell-cell communication (44); and Hox4a, which is a selective transcriptional regulator, were specifically upregulated on XCR1^+^ bystander dermal DCs.

Among the significantly downregulated genes were Vps39 and Clec16a, which are important for auto-lysosomal generation of self-antigens (45) and for surface expression of MHC-II molecules (46). Also, genes involved in TGF-β signaling required for Treg induction, such as *Icosl*, which is important for generating Foxp3^+^ Tregs (47); genes promoting Th2 such as *Bco1* (48); and genes blocking Th1 induction, such as *Tyrobp* (DAP12), which was shown to downregulate Th1 responses in intracellular infections (49–51). ATP synthesis pathways, which trigger IL-33 release and Th2 responses and subsequently block IL-12 production, were also inhibited as indicated by the downregulation of the ATP synthases mt-Atp6 and mt-Atp8 and of the cytochrome oxidases mt-Co2 and mt-Co3 (52, 53) (Figure 5C).

Together, our data provide a map of genes by which DC vaccine–activated MHC-II^hi^ bystander cDC1s acquire a broad phenotype on one hand toward Th1 induction, but on the other hand, away from a tolerogenic, self-antigen presenting, Treg-inducing or Th2-inducing gene signature.

### Bystander XCR1^+^ cDC1s downregulate MHC-II genes and acquire IL-12–producing potential.

**Figure 6:**
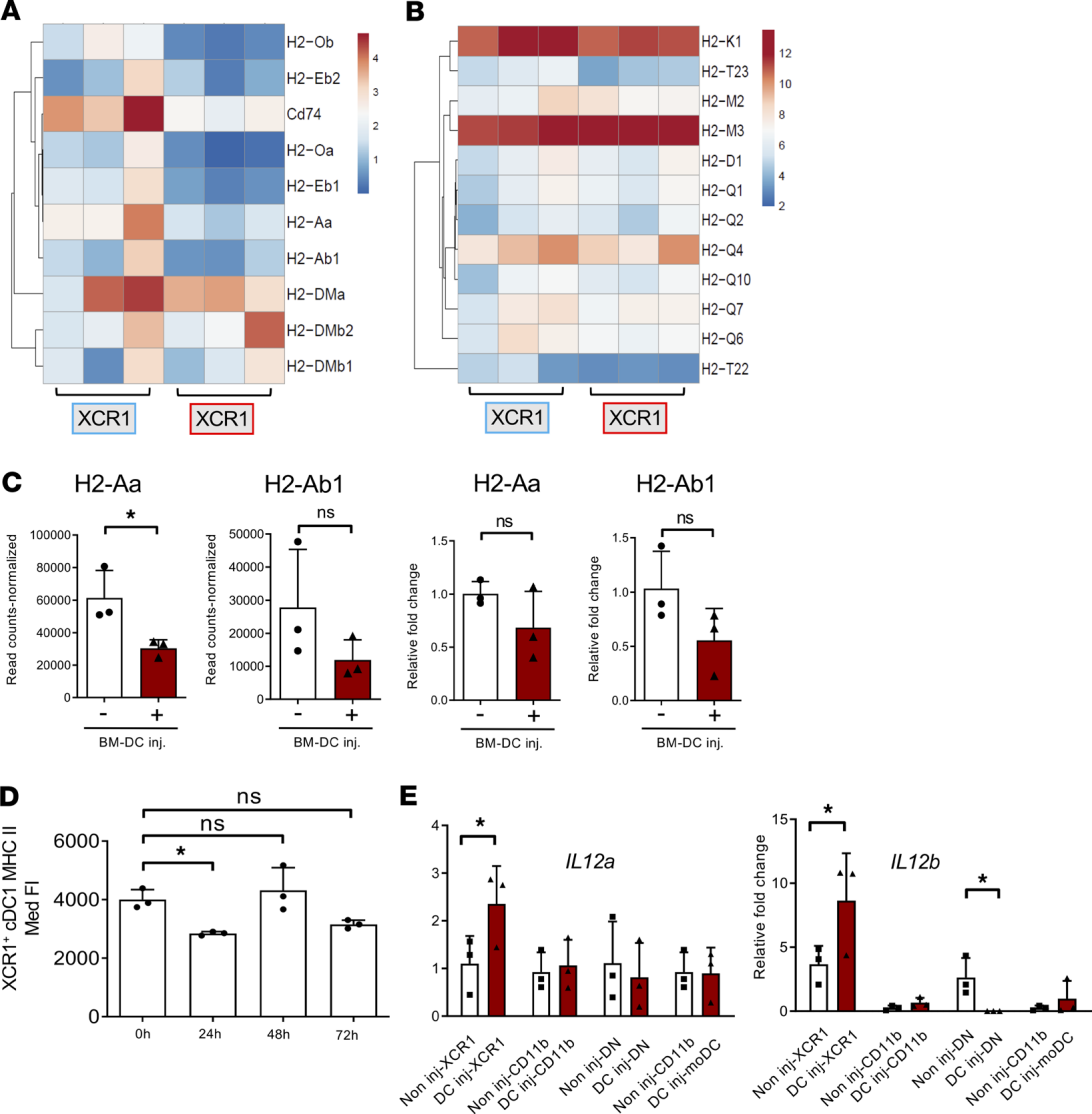
Loss of MHC-II but not MHC-I genes and acquisition of IL-12 production by bystander cDC1s. (**A** and **B**) Heatmap plots for the differential expression of MHC-II (left panel) and MHC-I (right panel) genes in the XCR1^+^CD11c^int^MHC-II^hi^ cDC1 subset before and 48 hours after immunization. (**C**) Normalized read counts (left panel) and relative fold change (right panel) by qPCR in the XCR1^+^ CD11c^int^MHC-II^hi^ cDC1 subset before and 48 hours after immunization. (**D**) Median fluorescence intensity of MHC-II on XCR1^+^CD11c^int^MHC-II^hi^ cDC1s before and 24, 48, 72 hours after BM-DC immunization. (**E**) qPCR analysis of *Il12a* and *Il12b* expression in all sorted DC subsets before and after 48 hours of immunization; *n* = 3; data represent mean ± SEM. (**C** and **E**) Unpaired, 2-tailed Student’s *t* test; *P < 0.05. (**D**) One-way ANOVA with multiple comparisons and Tukey’s post hoc test; *P < 0.05.

As indicated above, self-antigen presentation appears to be downregulated in bystander XCR1^+^ cDC1. More detailed analyses of MHC and related genes indicated a nonsignificant reduction of several MHC-II molecules (and also the invariant chain CD74) in bystander activated XCR1^+^ cDC1s on the transcript level (Figure 6, A and C). Such a reduction was not observed for classical and nonclassical MHC-I molecules (Figure 6B). Contrarywise, the MHC-II protein surface expression significantly reduced at an earlier time point after BM-DC injection (24 hours) and was significantly increased again after 48 hours (Figure 6D). This suggests a transfer of MHC molecules presenting the OVA antigen from the injected BM-DCs to the endogenous bystander DCs. The transfer of antigens between DCs has been shown before (54), but the exact mechanism in our setup needs further investigations.

*Il12a* and *Il12b* did not appear upregulated by XCR1^+^ dermal DCs in the RNA-seq analysis. Nevertheless, the upregulation of *Il12a* and *Il12b* on bystander-activated XCR1^+^ cDC1s was confirmed with real-time PCR, and both genes were found to be specifically upregulated on the designated XCR1^+^ subset and not on any of the other bystander-activated DC subsets (Figure 6E). We assume that not all endogenous XCR1^+^ DCs are activated to become bystander DCs, since they may carry out their tolerogenic functions (55). In addition, analyzing only 100 cells may not allow detecting enough low copy number genes, such as the IL-12a signal, and only strongly regulated genes become visible. Together, the transcriptional profiling of XCR1^+^ lymph node–resident bystander cDC1s is characterized by a downregulation of genes providing non-Th1 polarizing signals and the upregulation of genes such as *Il12a* and *Il12b* required for IL-12p70–mediated Th1 polarization.

## Discussion

DCs are the dominant immune cells to induce T cell priming in vivo. Also, the instruction of Th cell responses by DCs providing polarizing signals has become generally accepted. Among the Th1-instructing cytokines, IL-12p70 plays a prominent role. Here, we address whether the priming capacity and polarizing IL-12p70 signals are derived from injected vaccine DCs. We employed s.c. injection of BM-DCs into mice as a model to test different chimeric situations where injected BM-DCs and recipient mouse strains were bearing different genetic deficiencies. Our BM-DC vaccines are close to clinical studies with human MoDC vaccines, due to the fact that GM-CSF–generated BM-DCs are monocyte derived (17). The use of OVA antigen here may not allow simple extrapolation of our findings to human clinical studies using tumor-antigen pulsed MoDCs. However, our results using clear-cut genetic models to determine the source of IL-12 for Th1 induction may encourage similar investigations in human DC vaccine settings to improve vaccination success.

It has been observed before that priming of CD8^+^ CTL responses after virus infection or DC antitumor vaccines relies on antigen transport by migration of tissue-resident or injected DCs but that antigen presentation is largely dependent on lymph node–resident cDC1 or undefined endogenous DCs (56, 57). Similarly, our data reveal that s.c. injected vaccine BM-DCs only partially contribute to antigen presentation at an early stage (24 hours), and they do not contribute to Th1 polarization. A major part of antigen presentation for Th0 induction and the entire capacity for Th1 polarization is mediated by endogenous XCR1^+^ resident bystander cDC1s at later time points (48–72 hours). However, BM-DCs migration to the draining lymph node is strictly required, and bystander activation for IL-12 production seems to occur in the lymph node. Our findings argue for a step-wise process of priming naive T cells into an IL-2^+^IFN-γ^–^ Th0 phenotype by the injected DCs, followed by a communication between injected BM-DCs and XCR1^+^ bystander cDC1s. Bystander contact includes transfer of antigen and initiation of IL-12p70 production. This period is followed by contacts of activated IL-12p70^+^XCR1^+^ bystander cDC1s with the Th0 cells to continue antigen presentation and conversion into Th1 polarized cells. RNA-seq allowed the identification of transcriptional changes during the conversion of lymph node–resident XCR1^+^ cDC1s into bystander-activated cDC1s. Among those, DC genes known to polarize naive T cells into Treg or Th2/Th9 immune responses or to counteract IL-12 production were downregulated, while Th1 supporting genes were induced.

The optimization of DC vaccination protocols has focused mainly on enhancing the activation of generated DCs (58, 59), their cytokine production profile (60), and their migration capacity (61). Other studies attempted to combine the vaccine injection with adjusting the immunosuppressive milieu of the tumor microenvironment to a more immunogenic one — for example, by blocking inhibitory receptors such as PD-1/PD-L1 (62).

In this study, we found that endogenous DCs were critically required to induce polarized Th1 responses and enhance Th0 priming by vaccine DCs. We were able to identify XCR1^+^ lymph node–resident cDC1s as communication partners that take up the message delivered by the injected vaccine DC and are responsible for promoting full-blown Th1 responses. This opens up a potentially new level of complexity when considering strategies for vaccine DC optimization. The requirement of endogenous DCs for optimal antitumor DC vaccination is of clinical importance, since these patients are treated with immunosuppressive chemotherapy and are subjected to γ-irradiation that will affect endogenous DC populations. In contrast, the use of CTLA-4– and PD-1–targeted checkpoint inhibitors would not negatively affect endogenous DCs.

This study also sheds light on the question why the IL-1β/TNF-α/IL-6/PGE_2_ matured vaccine DCs are successful in Th1 priming, despite a lack of IL-12–producing capacity (29, 30). This was also reported for BM-DC immunization of *Leishmania major*–challenged mice, where IL-12 production by BM-DCs was not required and, rather, recipient IL-12 was mandatory. The recipient source of IL-12 was not identified (31). The same group showed that immunization with antigen-pulsed Langerhans cells strictly required their ability to produce IL-12 (63), indicating that the source of IL-12 required for optimal Th1 polarization changes depending on the DC subset. Although IL-12p70 production by vaccine DCs correlates with better immune responses in tumor patients (64–66), it remained open whether this vaccine DC–derived IL-12p70 promotes adaptive T cell responses or acts locally on innate NK cells, as observed in toxoplasma infection (67). Another clinical study shows a positive correlation between IL-12p70 production by the DCs before intranodal injection and the time to progression. However, the IL-12p70 production does not correlate with the IFN-γ ELISPOT responses of the CD4^+^ or CD8^+^ T cells of the patients (68). These findings also argue for a T cell–independent benefit of IL-12p70 production by the DCs for the patients. Of note, this intranodal injection does not require DC migration to the lymph nodes, and DCs may not have to decide between migration or cytokine secretion programs (32). Others found a positive correlation between IL-12p70 production by the DCs before i.v. injection and the time to progression, as well as the IFN-γ/IL-13 and IFN-γ/IL-5 ratios of CD8^+^ T cells, but not CD4^+^ Th1 (69). Thus, although the patients benefit from IL-12p70 production by vaccine DCs, a direct effect of vaccine-derived IL-12p70 on Th1 induction remains to be shown. Both studies (68, 69) used MoDCs, which are the human correlates to our murine BM-DCs (17) but differ from our setting by their intranodal or i.v. injection.

Other studies have shown IL-12–independent Th1 priming pathways, such as TNF receptor family members CD27 and OX40 on T cells interacting with their ligands CD70 and OX40L (70, 71). In our study, IL-12 production strongly supported Th1 polarization, and the XCR1^+^ migratory cDC1s were the mediators of this function. These cells appear to take over the Th1 polarization function from the injected BM-DCs at a later stage of the T cell response, when the initial antigen presentation phase is terminated and T cells enter their proliferative phase (42).

Interestingly, we also find that CD103^+^XCR1^+^ resident cDC1s were required for antigen presentation later in the T cell response, indicating antigen transfer from the injected BM-DCs. This is not caused by handover of antigen to CD103^+^XCR1^+^ migratory cDC1s in the skin, as observed for injected apoptotic DCs (72, 73), since no T cell priming or polarization occurred when antigen-loaded *Ccr7*^–/–^ BM-DC were used for immunization. Several mechanisms of antigen transfer between different DC subsets have been suggested — for example, via trogocytosis (74) or via exosomes (75) — and both pathways have been implicated in the transfer of peptide-bound MHC molecules and costimulatory molecules. Studies on viral models such as herpes simplex virus (HSV) have shown that viral antigens are handed over to XCR1^+^ lymph node–resident cross-presenting DCs for an optimum CTL response (56, 76). The requirement for endogenous DCs to support optimal CD4^+^ T cell responses by DC-DC contacts in lymph nodes has been observed before, but the endogenous DC subset was not identified and further bystander function for Th1 polarization was not investigated (77). Our data suggest that migrated BM-DCs transfer antigen and Th1 polarizing information specifically to XCR1^+^ bystander–activated DCs in the lymph node.

In the context of CpG adjuvanted immunization relying exclusively on endogenous DCs, large numbers of monocytes are mobilized to lymph nodes (78, 79), and MoDCs were identified as the major source of IL-12, supporting CD4^+^ and CD8^+^ T cell responses (80). We show that the XCR1^+^ DC subset is required for Th1 polarization, which highlights the importance of identifying the roles of different DC subsets depending on the context. Here, for CD4^+^ Th1 polarization, it comes as no surprise that XCR1^+^ cDC1s are the subset interacting with vaccine DCs, since they were shown to be the major IL-12–producing subset under Th1 priming conditions in different tissues (81, 82).

Here, we evaluated how the transcriptome of steady state MHC-II^hi^ cDC1s changes after generation of Th1 priming bystander DCs. During the steady state, MHC-II^hi^ DCs within lymph nodes represent tolerogenic migratory DCs (steady state migratory DCs; ssmDCs) that appear as a semimature stage with upregulated levels of nuclear RelB and surface MHC-II, CD86, CD40, and CCR7 molecules. However, all of these markers are expressed lower, as compared with pathogen- or inflammation-matured migratory DCs (5, 7, 83). In addition, ssmDCs express a TGF-β to induce Tregs, whereas pathogen-matured DCs produce proinflammatory cytokines to polarize CD4^+^ Th cell responses (33, 84). The dermal ssm-cDC1 subset, identified by expression of XCR1, CD103, and Langerin, has been characterized transcriptionally and revealed a matured phenotype with expression of RelB, IL-12p40, and CCR7 (84). Functionally, we found earlier that the ssm-cDC1s converted naive CD4^+^ T cells into Foxp3^+^ peripheral Tregs in a TGF-β–dependent manner in the skin draining lymph nodes (5). This TGF-β signature was later confirmed at the transcriptome level (84). We found that immunogenic bystander cDC1s did not markedly upregulate typical RNA signatures or markers for DC maturation; however, the transcriptional TGF-β signature decreased. This indicates that functionally tolerogenic XCR1^+^ ssm-cDC1s may be reprogrammed in the lymph node to become immunogenic bystander DCs. Functional plasticity of ssm-Langerhans cells had been shown before by their continued capacity to internalize antigens after migration into the draining lymph nodes (85). Our previous data show that BM-DCs that were matured with the inflammatory stimulus TNF maintained maturation plasticity, since they could be further stimulated by LPS in vitro to release IL-12p70 or in vivo by endogenous stimuli after s.c. injection to polarize for Th1 responses (33) instead of inducing tolerance by i.v. injection (86). Together, the transcriptional changes in bystander cDC1s indicate that they downregulate tolerogenic steady-state functions and become activated to induce Th1 responses. The data provide evidence that XCR1^+^ lymph node–resident cDC1s can undergo functional reprogramming and activation into mature bystander DCs by LPS from the migrated BM-DCs or other bystander signals sensed in the lymph node that appeared inflamed with increased cellularity.

Our data indicate that only IL-12 production by endogenous cDC1 in the draining lymph nodes, but not the injected DCs, instructs Th1 polarization. The exocytosis of IL-12 is mediated by the SNARE family member VAMP7 (87). Among the upregulated genes, we identified *Tmem79*, which is reported to be involved in exocytosis (44). It remains to be tested whether *Tmem79* also is involved in this process.

A significant increase in *IL12a* and *IL12b* gene expression by quantitative PCR (qPCR) that we detected specifically in XCR1^+^ migratory DCs after BM-DC immunization was not observed by RNA-seq. While the low number of DCs used for sequencing can attribute to such a discrepancy, it is also possible that the bystander activation signal was diluted by the remaining steady-state XCR1^+^ migratory cDC1s that still carry out their tolerogenic functions. Such a heterogeneity has been observed by single cell sequencing among LPS-stimulated spleen cells, where the DCs clustered differently when compared with the existing marker-based classification (88). Using our defined transcriptional signature for bystander-activated XCR1^+^ migratory cDC1s, it might be possible to distinguish them from their steady-state counterpart and specifically target them for enhancing DC vaccination protocols.

In conclusion, our data suggest that Th0 priming strictly requires the vaccine DCs but endogenous bystander DCs for IL-12p70 production and Th1 polarization in the OVA/OT-II model (Supplemental Figure 4). Only the CCR7^–^ resident, and not the migratory fraction, of the XCR1^+^ cDC1 subset acquired bystander function. Although DC phenotypes and functions in the lymph nodes may change in mice with established tumors or ongoing infections, these findings may be of translational importance but require confirmation in mouse tumor models and human DC vaccination studies in immunocompromised tumor patients.

## Methods

Supplemental Methods are available online with this article.

### Study approval

All animal experiments were performed according to the German animal protection law, as well as after approval and under control of the local authorities.

## Author contributions

MBL and FE designed the experiments; DA, PA, and VP conducted experiments and acquired data; DA, PA, KUF, and FE analyzed data; AB and TK provided reagents; and DA and MBL wrote the manuscript.

## Supplementary Material

Supplemental data

## Version 1 05/21/2020

Electronic publication
