# Supplementary material for: IL-12 from endogenous cDC1, and not vaccine DC, is required for Th1 induction
Source: JCI Insight. 2020 May 21;5(10):e135143. doi: 10.1172/jci.insight.135143 (PMC7259537; doi:10.1172/jci.insight.135143)
Supplement: Supplemental data [file jciinsight-5-135143-s158.pdf]

Figure S1

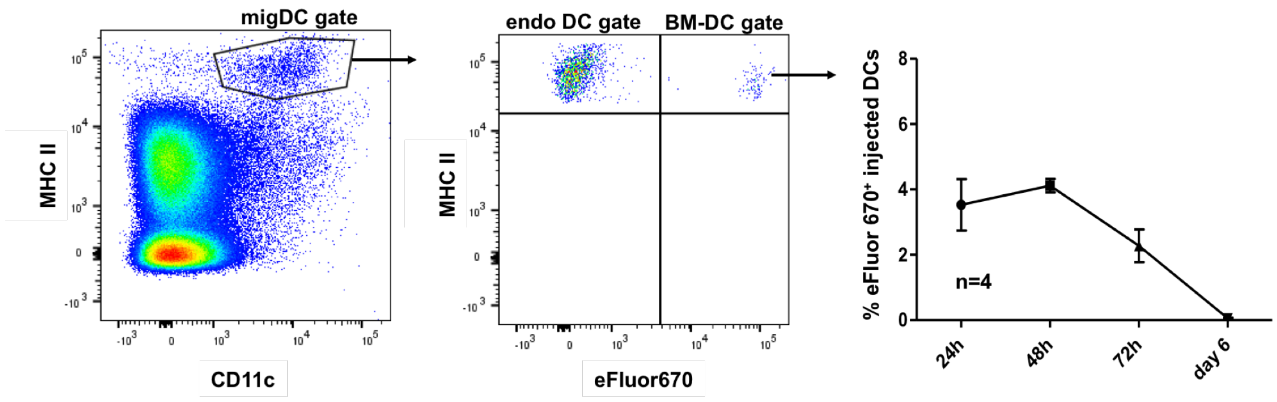

Figure S1 : **BM-DC label stability in vivo.** BM-DCs were labeled with eFluor670 and then injected s.c. into recipient mice and stained as indicated for FACS analysis. eFluor670^{+} BM-DCs were readily detectable for 72h but the label disappeared after 6 days. n= 4 mice

Figure S2

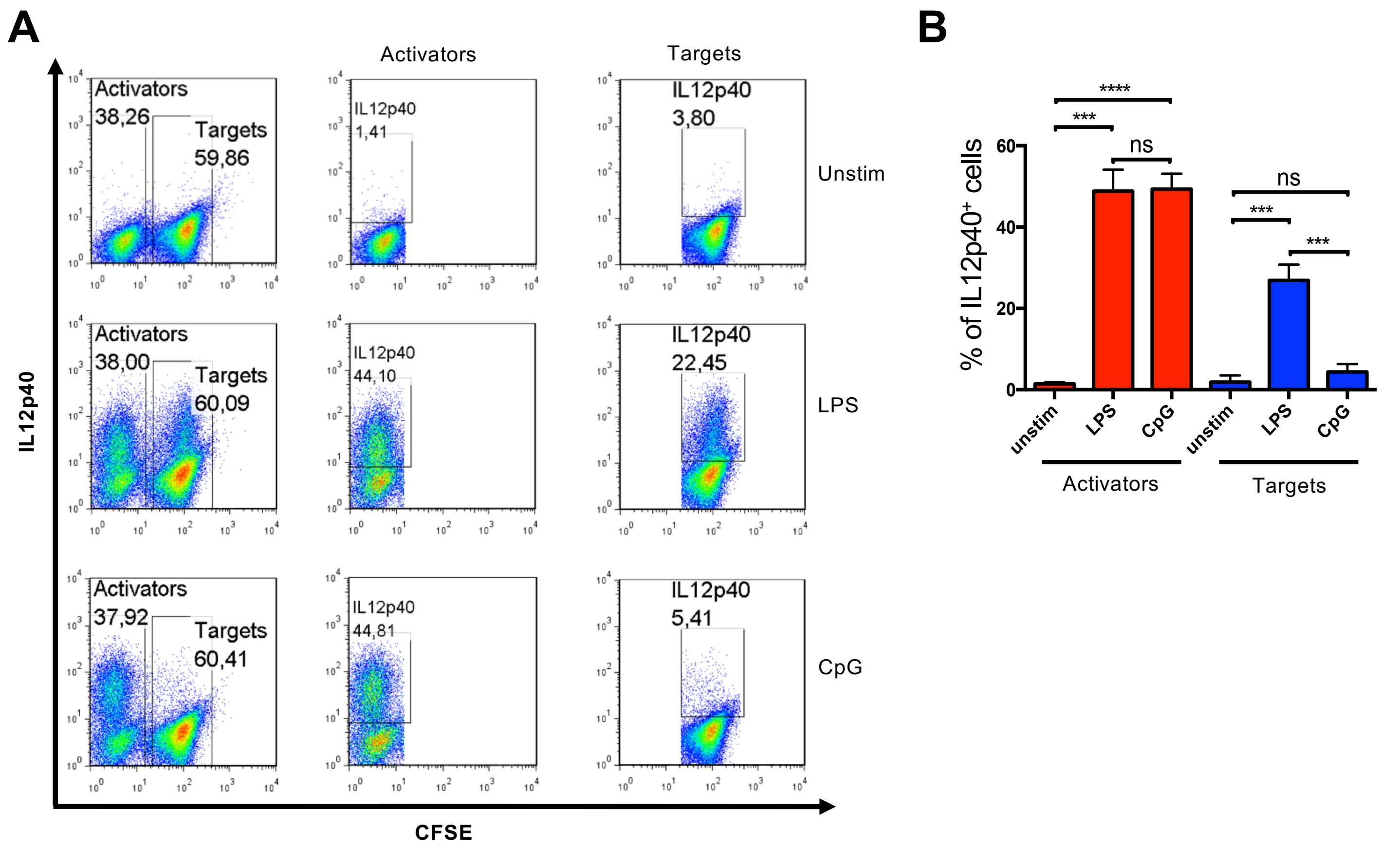

Figure S2: Production of IL-12p40 in trans may occur by LPS but not CpG induced BM-DC maturation. BM-DCs were matured with LPS, CpG or unstimulated for 6h, washed and co-cultured with CFSE-labeled immature BM-DCs, after another 16h cells were stained for intracellular IL-12p40 production. **A.** Representative flow cytometry plots for $CFSE^-$ donor DCs (Activiators) and $CFSE^+$ bystander activated DCs (Targets) and the % of IL-12p40 producing cells upon using untreated (upper), LPS-matured (middle), and CpG-matured (lower) Activator DCs **B.** Graphs showing the % of IL-$12p40^+$ cells from untreated, LPS or CpG-matured activator DCs and their respective bystander activated target DCs in three independent experiments. ****P < 0.00001 ***P < 0.0001, **p < 0.001, *p < 0.05.

Figure S3

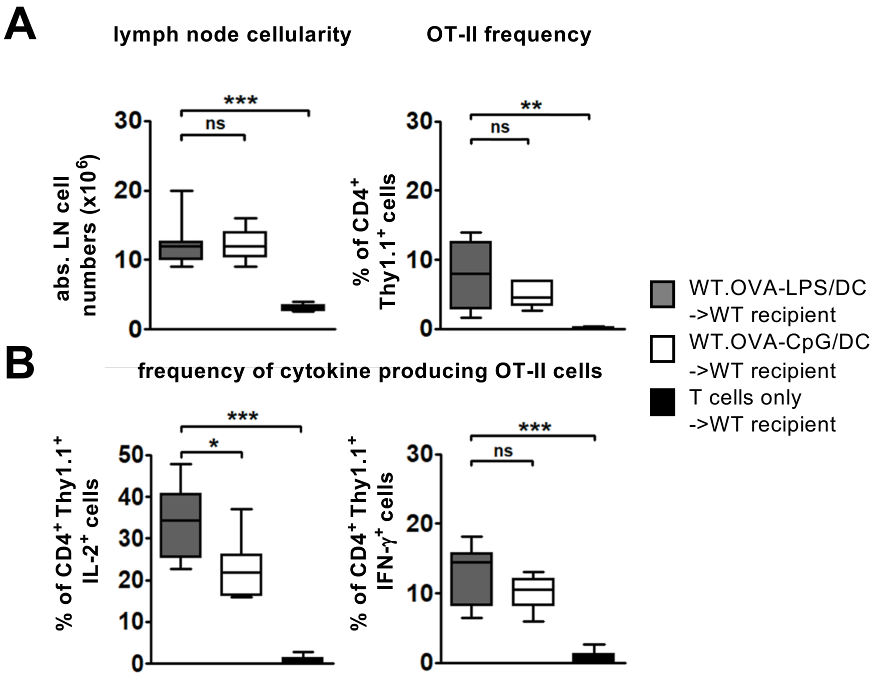

Figure S3 : LPS- or CpG-matured BM-DCs induce similar lymph node swelling, T cell expansion and Th1 polarization. Graphs comparing lymph node cell counts, frequency of injected OT-II^{+}.Thy1.1^{+}CD4^{+} T cells (**A)** and percentage of the cytokine producing cells **(B)** after s.c. injection of WT.OVA-LPS/DC (grey bars) or WT.OVA-CpG/DC (white bars) compared to T cell injection with no BM-DC injection (black bars) into C57BL/6.WT recipient mice. Data are representative of three independent experiments analyzing at least 5 mice per group. ***P < 0.0001, **p < 0.001, *p < 0.05.

Figure S4

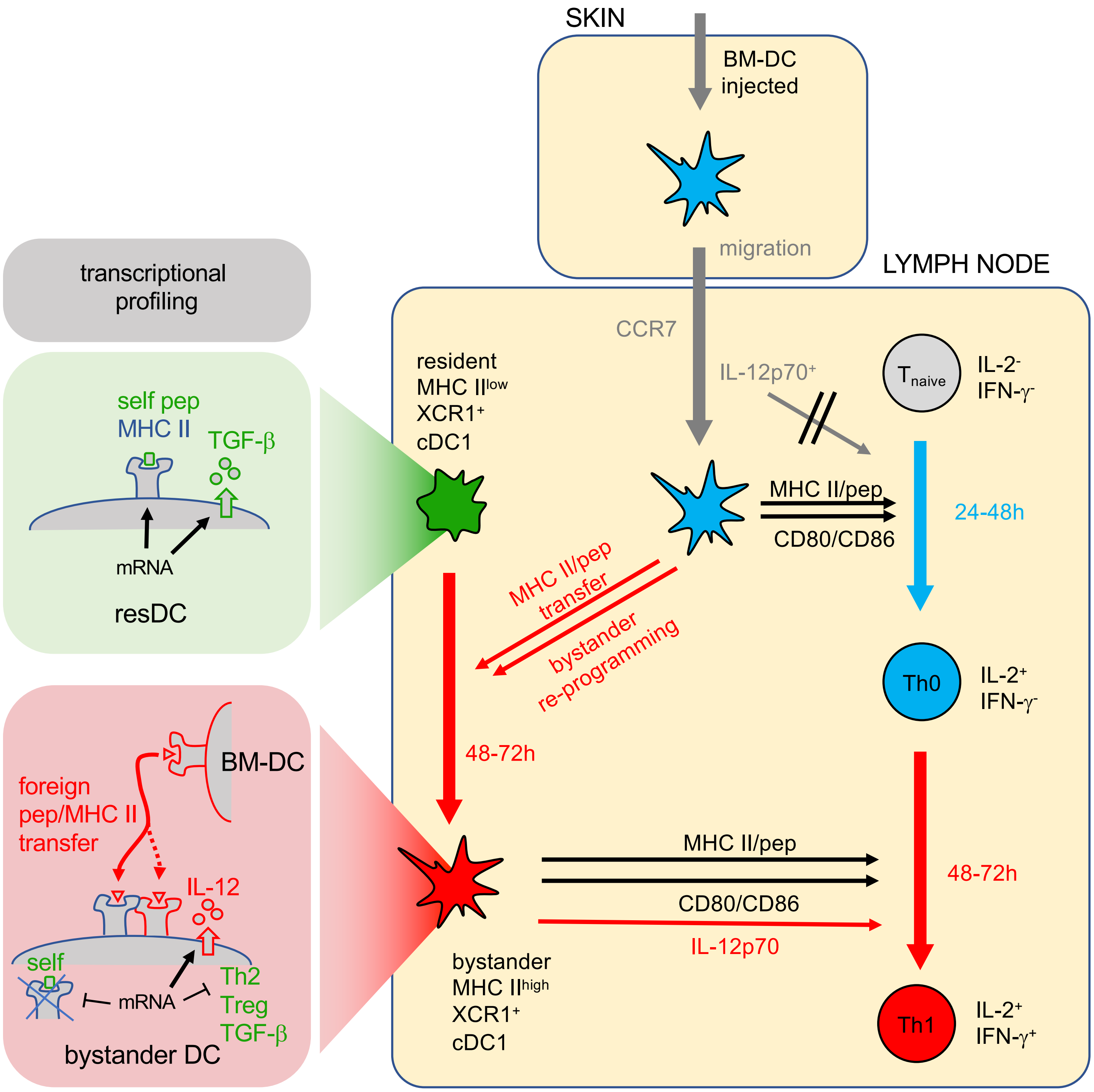

Figure S4: **A model for the functional switch in XCR1^{+} DCs following BM-DC injection.** The antigen-loaded matured and injected BM-DC (blue DC) migrates to the lymph node and provides the first two signals to the naive T cell (grey T cell) during the early stage of the immune response (24-48h). As a result, the T cell converts to an IL-2^{+} IFN-γ^{-} Th0 stage (blue T cell). This is followed by MHC II molecule/antigen transfer and bystander re-programming lymph node-resident XCR1+ cDC1 (green DC) from a self-antigen presenting mode and TGF-β cytokine profile into a matured XCR1+ bystander cDC1 (red DC) that is contributing to antigen presentation, shows a IL-12p70^{+} cytokine profile, but signatures for self-antigen presentation, Th2 and Treg inducing and the TGF-β are down-regulated.

Methods

Mice

C57BL/6 were purchased from Charles River (Sulzfeld, Germany), OT-II mice were kindly provided by Francis Carbone, Melbourne, Australia and were crossed with congenic C57BL/6 Thy1.1 mice. *Il12a*$^{-/-}$ (1) *and* Yet40 reporter mice (2) *were kindly provided by Gottfried Alber*. *Ccr7*$^{-/-}$ mice (3) were obtained from Martin Lipp and Reinhold Förster. *Il12a.Ccr7*$^{-/-}$ were bred in house. The B6.Cg-Xcr1$^{tm2(HBEGF/Venus)Ksho}$ mice were provided by the RIKEN BRC through the National BioResource Project of the MEXT/AMED, Japan (4), and B6.129S2-*H2*$^{dlAb1\text{-}Ea}$/J (MHC II$^{-/-}$) mice (5) were provided by Andreas Beilhack. All mice were bred in our own animal facilities at Würzburg, kept under specific pathogen-free conditions, and used at an age of 6 to 12 weeks.

Flow cytometry

Surface staining: performed at 4˚C using PBS supplemented with 0.1% BSA and 0.1% sodium azide (FACS buffer). mAbs were purchased from BioLegend, unless otherwise indicated. The murine antibodies used: CD11c-PECy7 (N418), MHC II-Alexa Fluor700 (M5/114.15.2) CD11b-PerCP-Cy5.5 (M1/70), B220-Pacific Blue or FITC (RA-3-6B2), CD4-APC or FITC (GK 1.5), CD3-FITC (145.2C11), XCR1-APC (ZET), CD103-biotin (2E7), Thy1.1-PerCP-Cy5.5 (OX-7). If primary antibodies were biotinylated, the following secondary antibodies were used: streptavidin–PECy7, or streptavidin–Brilliant Violet 510 (Biolegend). Fc receptors were blocked by pre-incubating the cells with 10% of supernatant from the 2.4G2 hybridoma (anti-FcγRII/III; ATCC) prior to staining. Intracellular staining: cells were fixed using 2% formaldehyde, permeabilized with perm buffer (0.5% saponin in FACS buffer) and stained at 4˚C using the following Abs in perm buffer: IFN-γ-PE (XMG1.2), IL-2-PE (JES6-5H4). Samples were measured in LSR II flow cytometer (BD Biosciences) and data were analyzed with FlowJo software v8.8.7 (TreeStar).

Generation of murine BM-DCs

BM-DCs were generated as previously described (6). Briefly, bone marrow precursor cells were obtained by perfusion from mice femurs and tibias and seeded in petri dishes (100 mm, Greiner) at 3x10^6 cells/dish in 10 ml of complete medium: RPMI 1640 (Sigma) supplemented with 10% heat-inactivated FCS (GIBCO), 100 U/ml penicillin (Sigma), 100 µg/ml streptomycin (Sigma), 2 mM L-glutamine (Sigma) and 50 µM β-

mercaptoethanol (Sigma-Aldrich). To induce the differentiation to DCs, the medium was additionally supplemented with 10% of a supernatant obtained from the X-63 cell line transfected with the GM-CSF murine gene. The cells were culture at 37°C, 7% CO_2. On day 3 of culture, 10 ml of complete RPMI medium supplemented with 10% GM-CSF-containing supernatant was added. On day 6, 10 ml of the medium was removed, and fresh medium was added as at day 3. The non-attached cells were harvested and used at day 8.

Lymph node and spleen cell preparation

Skin-draining lymph nodes (popliteal, inguinal, and cervical) and spleens, were cut into small pieces using forceps and digested for 20 min at RT with 1 mg/ml DNase I (Roche) and 1 mg/ml collagenase IV (Worthington) in the medium described above. To disrupt multicellular complexes that include DCs and T cells, 0.01 mM EDTA (Sigma) was added and incubation at RT was extended for 5 min. Cells were resuspended in ice cold PBS containing 5% FCS and 1 mM EDTA, transferred through a 70 µm cell strainer (BD) and counted. Splenic cells were additionally subjected to erythrocyte lysis by osmotic shock before enzymatic digestion.

DC vaccination

BM-DCs at day 8 of culture were matured with 0.5 µg/ml LPS or CpG (5 nmol/ml) for 4h, washed extensively and $2x10^6$ DCs injected into the footpad or into the flanks of mice. Popliteal or Inguinal lymph nodes were collected at the given time points, respectively. In case of adoptive OT-II T cell transfer, the BM-DCs were also loaded with 10 µM OVA peptide$_{327-339}$ and, one day before, single cell suspensions of lymph nodes (pooled skin-draining and mesenteric lymph nodes) and spleens from OT-II$^+$.Thy1.1$^+$ mice were prepared as described above and injected as bulk populations. Cells were labeled with CellTrace™ Violet Cell Proliferation dye and $1x10^7$ cells were injected into the lateral tail vein of recipient mice.

Ex vivo stimulation and cytokine staining of cells from lymph nodes and spleen

Single cell suspensions from skin-draining lymph nodes or from spleen were prepared as described above and resuspended at a density of $2x10^6$ cells per ml in complete RPMI 1640 medium. Cells were then stimulated with 30 ng/ml PMA (Sigma) and 1 µg/ml ionomycin (Sigma) in the presence of 5 µg/ml brefeldin A (Sigma) for 4 h at 37°C.

After stimulation, cells were stained for surface markers (CD4, Thy1.1), fixed in 2% formaldehyde for 20 min at RT, and stained intracellularly for IL-2 and IFN-γ in Permeabilization Buffer (eBioscience™) (PBS supplemented with 0.5% saponin, 0.1% BSA, and 0.05% sodium azide) for 45 min at 4°C.

Depletion of XCR1$^+$ DCs

For depletion of XCR1$^+$ DCs, transgenic mice were treated with 0.5 µg diphteria toxin (DTX) i.p. (Merck Millipore) on d-1, d0, followed by 0.25 µg DTX i.p on d3 and d5. Popliteal lymph nodes were collected on d6.

Immunofluorescence staining

For detection of IL-12p40-YFP, popliteal lymph nodes were fixed with periodate-lysine-paraformaldehyde fixative (PLP) at 4°C overnight and were passed through a 10% sucrose/PBS then 20% then 30% gradient. Lymph nodes were then frozen in tissue-tek (Sakura). Endogenous avidin and biotin in lymph node sections were blocked using Avidin/Biotin Blocking Kit (Vector Labs) and sections were stained using 2% FCS/PBS-diluted antibodies for B220-Alexa fluor 647 (RA-3-6B2), and Thy1.1-Biotin (HIS51) or CD11b-Biotin (M1/70) or XCR1 (ZET) respectively, followed by streptavidin–Cy3 (Biolegend). Injected BM-DCs were labeled with CellTrace™ Violet. Imaging was performed using LSM 780 (Carl Zeiss Microimaging). Quantitative image analysis was done using the Imaris software tools. The different labeled cells were localized using Imaris spot function and the relative distance was calculated using Excel software calculating the minimal distance in the X and Y planes.

RNA sequencing of lymph node DC subsets

Mice were injected with LPS/BM-DCs into the footpad and the popliteal and inguinal lymph nodes were collected after 48h. T and B cells were depleted by Dynabeads™ Biotin Binder (Invitrogen™) using Biotin-B220, CD3, and CD4. The negatively selected cells were sorted for different CD11c^{int} MHC II^{high} DC subsets using a BD FACS Aria III (precision: single-cell; nozzle: 100 µm). 100 cells for every population for in total 3 replicates were sorted into individual wells of a 96-well plate (Brand) filled with 4 µl lysis buffer (Takara). Cells were spun down, immediately chilled to 4°C and stored at −80°C. All the following experimental steps were performed using the SMART-Seq® v4 Ultra® Low Input RNA Kit (Takara) with a quarter of the recommended reagent

volumes. The PCR amplification was performed according to the manual using 21 cycles. Libraries were quantified by QubitTM 3.0 Fluometer (ThermoFisher) and quality was checked using 2100 Bioanalyzer with High Sensitivity DNA kit (Agilent). 0.5 ng of each library was subjected to a tagmentation-based protocol (Nextera XT, Illumina) using a quarter of the recommended reagent volumes, 10 min for tagmentation at 55°C and 1 min extension time during PCR for multiplexing. After PCR, the libraries were purified using AMPure XP beads and eluted in 15 µl of resuspension buffer. Libraries were pooled and sequenced in paired-end mode on the NextSeq500 sequencer (IIlumina) using the Mid Output 2×75 cycle kit.

Data analysis

Base calling was done by the internal software of the NextSeq 500 sequencer "NextSeq Control/RTA v2” and bcl2fastq2 Conversion Software v.2 was used to demultiplex the pooled libraries and to convert the bcl files generated by the sequencer to standard fastq files for downstream analysis. The generated raw reads were processed using FastQC 0.11.6 (http://www.bioinformatics.babraham.ac.uk/projects/fastqc) for assessing read quality, number of duplicates and presence of adapter sequences. After this, the Illumina Nextera adaptors were cleaved using cutadapt (7) (version 1.16) and resulting reads were further trimmed keeping a quality drop value below a mean of Q20. Further, the processed sequences were mapped to the mouse genome using the short-read aligner STAR (8) (version-2.5.2b) with genome and annotation files retrieved from GENCODE (July 2017, GRCm38.p5, M16). For all the studied samples, the proportion of reads uniquely mapped to the mouse reference genome ranged between 83% and 87% in total. The sequences aligning to specific genes were quantified using bedtools' (9) subcommand intersect (version 2.15.0). Next, the differentially expressed genes were identified using DESeq2 (10) (version 1.18.1). Only the genes having a Benjamini-Hochberg corrected p-value below 0.05 were classified as significantly differentially expressed (DEGs). The RNA-Seq data presented in this work has been deposited at the NCBI Gene Expression Omnibus (11) and can be accessed through GEO series accession number GSE124677.

Pathway analysis

Enrichment scores for pathways up or down-regulated by endogenous CD11c^{int} MHC II^{high} XCR1^{+} DCs was calculated using GOrilla analysis tool (Gene Ontology enRIchment anaLysis and visuaLizAtion tool). The data were plotted as FDR q-value after Benjamini and Hochberg correction of p-values. The threshold of significance was set at FDR q-value (0.05).

Quantitative real-time PCR.

cDNA libraries generated by the SMART-Seq® v4 Ultra® Low Input RNA Kit from the sorted endogenous CD11c^{int} MHC II^{high} DC subsets were used. Quantitative real-time PCR was performed on cDNA using iTaq™ Universal SYBR® Green Supermix (Bio-Rad). Reactions were run on a real-time quantitative PCR system (Roche, LightCycler® 96) and 18S was used as housekeeping gene. Relative expression differences were calculated using the ΔΔCt method (12). Primer sequences were as follows :*18S*; Fwd: 5′-GTAACCCGTTGAACCCCATT-3' and Rev: 5′-CGCTACTACCGATTGGATGG-3'. *Il12a*; Fwd: 5′-AGCTCCTCTCAGTGCCGGTC-3' and Rev: 5′-GGTCTTCAGCAGGTTTCGGG-3'. *Il12b*; Fwd: 5′-AGCAGTAGCAGTTCCCCTGA-3' and Rev: 5′AGTCCCTTTGGTCCAGTGTG-3'. *H2-Aa*; Fwd: 5′-GAGTCACACCCTGGAAAGGA-3' and Rev: 5′-GTTGGGGGTCaCTTGAAGAA-3'. *H2-Ab*; Fwd: 5′-TCTGCTCCGAATTCCTGACT-3' and Rev: 5′TCACAAGAGCTGAGGTGGTG-3'.

Statistics

Statistical analyses were performed using Prism 6.0 software (GraphPad Prism) for Fig 6C and 6E with unpaired, two-tailed Student's t-test. For all other graphs one-way ANOVA with multiple comparisons and Tukey's post-test. (*p < 0.05, **p < 0.01, ***p <0.005, ****p < 0.0001).

References

1. Mattner F, Magram J, Ferrante J, Launois P, Di Padova K, Behin R, et al. Genetically resistant mice lacking interleukin-12 are susceptible to infection with Leishmania major and mount a polarized Th2 cell response. *Eur J Immunol.* 1996;26(7):1553-9.
2. Reinhardt RL, Hong S, Kang SJ, Wang ZE, and Locksley RM. Visualization of IL-12/23p40 in vivo reveals immunostimulatory dendritic cell migrants that promote Th1 differentiation. *Journal of immunology.* 2006;177(3):1618-27.

3. Förster R, Schubel A, Breitfeld D, Kremmer E, Renner-Muller I, Wolf E, et al. CCR7 coordinates the primary immune response by establishing functional microenvironments in secondary lymphoid organs. *Cell.* 1999;99(1):23-33.
4. Yamazaki C, Sugiyama M, Ohta T, Hemmi H, Hamada E, Sasaki I, et al. Critical roles of a dendritic cell subset expressing a chemokine receptor, XCR1. *Journal of immunology.* 2013;190(12):6071-82.
5. Madsen L, Labrecque N, Engberg J, Dierich A, Svejgaard A, Benoist C, et al. Mice lacking all conventional MHC class II genes. *Proceedings of the National Academy of Sciences of the United States of America.* 1999;96(18):10338-43.
6. Lutz MB, Kukutsch N, Ogilvie AL, Rossner S, Koch F, Romani N, et al. An advanced culture method for generating large quantities of highly pure dendritic cells from mouse bone marrow. *J Immunol Methods.* 1999;223(1):77-92.
7. Martin MJEj. Cutadapt removes adapter sequences from high-throughput sequencing reads. 2011;17(1):pp. 10-2.
8. Dobin A, Davis CA, Schlesinger F, Drenkow J, Zaleski C, Jha S, et al. STAR: ultrafast universal RNA-seq aligner. *Bioinformatics (Oxford, England).* 2013;29(1):15-21.
9. Quinlan AR, and Hall IM. BEDTools: a flexible suite of utilities for comparing genomic features. *Bioinformatics (Oxford, England).* 2010;26(6):841-2.
10. Love MI, Huber W, and Anders S. Moderated estimation of fold change and dispersion for RNA-seq data with DESeq2. *Genome biology.* 2014;15(12):550.
11. Edgar R, Domrachev M, and Lash AE. Gene Expression Omnibus: NCBI gene expression and hybridization array data repository. *Nucleic Acids Res.* 2002;30(1):207-10.
12. Livak KJ, and Schmittgen TD. Analysis of relative gene expression data using real-time quantitative PCR and the 2(-Delta Delta C(T)) Method. *Methods.* 2001;25(4):402-8.
